# Recent Developments of an Opto-Electronic THz Spectrometer for High-Resolution Spectroscopy

**DOI:** 10.3390/s91109039

**Published:** 2009-11-13

**Authors:** Francis Hindle, Chun Yang, Gael Mouret, Arnaud Cuisset, Robin Bocquet, Jean-François Lampin, Karine Blary, Emilien Peytavit, Tahsin Akalin, Guillaume Ducournau

**Affiliations:** 1 Université Lille Nord de France, F-59000 Lille, France; E-Mails: jean-francois.lampin@isen.iemn.univ-lille1.fr (J.F.L.); Karine.Blary@IEMN.Univ-Lille1.fr (K.B.); emilien.peytavit@iemn.univ-lille1.fr (E.P.); Tahsin.Akalin@IEMN.Univ-Lille1.fr (T.A.); Guillaume.Ducournau@iemn.univ-lille1.fr (G.D.); 2 ULCO, LPCA, 189A Av. Maurice Schumann, F-59140 Dunkerque, France; E-Mails: chun.yang@univ-littoral.fr (C.Y.); Gael.Mouret@univ-littoral.fr (G.M.); arnaud.cuisset@univ-littoral.fr (A.C.); bocquet@univ-littoral.fr (R.B.); 3 CNRS, UMR 8101, F-59140 Dunkerque, France; 4 IEMN, Avenue Poincaré, BP 60069, F-59652 Villeneuve d'Ascq cedex, France; 5 CNRS, UMR 8520, F-59652 Villeneuve d'Ascq, France

**Keywords:** THz, photomixing, high-resolution spectroscopy, gas phase

## Abstract

A review is provided of sources and detectors that can be employed in the THz range before the description of an opto-electronic source of monochromatic THz radiation. The realized spectrometer has been applied to gas phase spectroscopy. Air-broadening coefficients of HCN are determined and the insensitivity of this technique to aerosols is demonstrated by the analysis of cigarette smoke. A multiple pass sample cell has been used to obtain a sensitivity improvement allowing transitions of the volatile organic compounds to be observed. A solution to the frequency metrology is presented and promises to yield accurate molecular line center measurements.

## 1. Introduction

The Terahertz (THz) frequency domain is often referred to as the range from 100 GHz to 10 THz and is also variously known as the sub-mm or far-infrared region, generally depending on the techniques employed for the generation of the radiation. Compared to other frequency regimes the THz region has suffered from a significant lack of technological development and so this part of the electromagnetic spectrum is only presently being explored and applications identified. For many applications the attraction of the use of THz radiation is due to its transition position, being able to provide detailed molecular information and easily penetrate normally diffusive materials. The most well known THz application that has been identified is the security screening of airline passengers, however, the astrophysics community has quietly and successfully utilized THz radiation for a number of decades during which it has provided significant information such as the visualization of objects hidden behind interstellar dust clouds. The continued development of new and existing applications is dependent on the availability of powerful sources that can cover the entire range, and sensitive detectors with short response times at room temperature.

### 1.1. Sources

The lack of powerful sources that operate at THz frequencies is due to the technological difficulties in their construction, related once again to the position of THz radiation in the electromagnetic spectrum. On the lower frequency side microwave sources are readily commercially available and can easily provide a powerful, narrow line width, tunable source around 20 GHz. At frequencies above 100 GHz it becomes increasing difficult to produce electronic devices with a sufficiently short carrier transit time, hence only small devices can be used, which provide limited output powers. On the optical or high frequency side infrared lasers are easily manufactured from semiconductor materials to form an easy to use device with reasonable power levels, but the interband concept cannot be exploited at THz frequencies as no semiconductor materials with a sufficiently narrow bandgap are available. Despite these difficulties, a small number of sources are capable of directly producing THz radiation.

Backward Wave Oscillators (BWO) or Carcinotrons use the interaction between a electron beam and a slow wave structure to produce radiation at frequencies up to 1.5 THz with 1 mW of power. Spectral purities as narrow as 50 kHz can be achieved, but a single device can only cover part of the THz range and has a relatively short lifetime. The number of commercial device manufacturers is very limited due to the expense of the fabrication and limited demand. Synchrotrons and free electron lasers also fall in the category of direct generation as they can provide intense radiation over the entire band. These sources are massive installations that are extremely expensive to construct and operate, and so they remain the domain of national and international science programs [1,2]. Molecular lasers also provide access to many laser lines in the THz range. Alone they are little use for spectroscopy as they operate at fixed frequency, however a tunable sideband can be generated by the addition of a microwave mixing stage giving access to all frequencies within 20 GHz of the carrier [3,4]. This solution also benefits from the possibility of using a heterodyne measurement configuration avoiding the requirement for a cryogenically cooled detector. The most recent addition to the family of direct sources is the Quantum Cascade Laser (QCL) which has developed rapidly since 1994 thanks to molecular beam epitaxy and metalorganic vapour phase epitaxy that allow the fabrication of quantum hetrostructures. A device is built up of thin periodic layers of varying composition to form a superlattice resulting in the creation of a series of discrete electronic subbands [5]. A stairway of subbands allows a single electron to cascade down generating multiple photons. The device emission wavelength is dependent on the layer thicknesses rather than the bandgap of the material, operation at 2.5 THz has demonstrated the utility of QCL for measurement of a transition of CH_3_OH [6]. Although they can produce several mW of power their shortcomings are the limited tuning range and the need for cryogenic cooling. They are however ideal candidates for the local oscillators required to construct heterodyne instruments for astronomy.

Owing to the limited availability of direct sources much effort has been focused on frequency conversion techniques whereby an intense source in a neighbouring band is used as the primary source. The frequency multiplication of microwave sources is particularly attractive because a tunable source with a spectral purity better than 10 kHz can be achieved. The key elements are the frequency multiplier stages that are used to produce the high order harmonics required and considerable success has been realized using this technique for astrophysics applications. Indeed this approach resulted in the first coherent source above 1 THz [7]. The technological developments that have assisted this continued progress are the use of monolithic microwave integrated circuit (MMIC) devices, and the fabrication of precision THz waveguides by computerized milling machines (CNC). Frequency multiplication is presently able to operate at 1.9 THz providing in the order of 10 μW of power when cooled to cryogenic temperatures. The limitations of this solution are the tunablity of a given multiplier chain that is often in the region of 10% and the availability of the components [8]. An alternative to frequency multiplication is down conversion from the optical band. Progress in the elaboration of crystals and short-lifetime semiconductor materials coupled with the ultra-rapid optical pulses available from mode-locked Ti:Sa lasers has provided numerous solutions for the generation and detection of broadband THz pulses. This kind of source with a coherent detection scheme can be employed to form a Time Domain Spectroscopy instrument giving access to the range 0.3 to 3 THz with a spectral resolution in the order of 1 GHz and is generally suitable for the spectroscopy of solid and liquid phase materials [9,10]. At present such THz time domain spectrometers are widely used to study low quality factors resonance features. Spectral resolutions approximately three orders of magnitude narrower can be obtained by the optical heterodyning of two continuous-wave (cw) lasers. A THz beatnote in the optical domain is converted into THz radiation by a semiconductor material with a short charge carrier lifetime. This technique, also known as photomixing, has the advantage of providing access to frequencies from 0.3 to 3 THz and its tuning is solely dependent on the source lasers. Its disadvantage is the available power which is presently limited to values around 1 μW. The attractive spectral resolution and large tunability of this source has ensured its utility for the spectroscopy of gases.

### 1.2. Detectors

Detecting continuous-wave THz radiation with high signal/noise and large bandwidth is also problematic. Historically, the first sensitive detectors in this band were thermal, and are still used because they are simple, have an unrivalled spectral band and can be very sensitive [11]. They are constituted of a thermally isolated THz absorbing material in contact with a thermometer, in fact the absorber and the thermometer are often the same material. The chief difference between the different kinds of detectors is the mechanism used to measure the temperature of the absorber. It can be the change of a resistance (bolometer), a thermoelectric effect (thermopile), a variation of spontaneous polarization (pyroelectric detector) or the dilatation of a gas (Golay cell). All these principles may be used for room temperature THz detection providing a noise equivalent power (NEP) generally in the range 10^−6^–10^−10^ W/√Hz. It should be mentioned that commercial detectors are generally not optimized for the THz or far-infrared range but rather for the mid- and near-infrared. In the 60s the cryogenic cooling of bolometers was investigated, and sensitive and reliable cryogenic THz bolometers were obtained with semiconductors like silicon and germanium. The typical NEP is generally close to 10^−12^ W/√Hz. Bolometers are not only well suited to detection of CW THz signals but also wide bandwidth signals like black-body emission or THz pulses.

Another principle that can be used in THz detectors is the photoconductive effect in semiconductors. These detectors need cooling at liquid helium temperature or below due to the low energy of THz photons (1–10 meV). Impurity photoconductors (like doped germanium) are usable above 3 THz and free-carrier photoconductors (InSb) are usable below, the NEP realized is generally close to 10^−12^ W/√Hz. The advantage of photoconductors compared to bolometers is that they have a fast reponse time (μs compared to ms). In the context of gas spectroscopy, this can be a real advantage when used in conjunction with a frequency modulated THz source.

The last principle used in THz detectors is based on non-linear conversion processes. This technique initially originates from microwaves and has been extensively used in the millimetric and sub-millimetric range. A low-capacitance diode with a non-linear *I*(*V*) curve is used as a quadratic detector. When a THz alternative voltage is applied at the terminals of the diode a dc voltage proportional to the THz power is generated (video or direct detection). Low barrier Schottky diodes are generally used for this purpose. The NEP is close to 10^−12^ W/√Hz near 100 GHz and 10^−10^ W/√Hz near 1 THz. The advantage of this detector is a good sensitivity for a room-temperature device, the disadvantage is that it needs an impedance matching to achieve these values and the bandwith is generally limited (typically 1.5:1 for a detector in metallic rectangular waveguide). It is also possible to convert the THz frequency to non-zero frequency—called intermediate frequency (IF)—by using a powerful tunable THz source (local oscillator) in addition to the THz wave to detect. This technique called heterodyne mixing is not easy to apply at THz frequencies because of the lack of convenient sources. Molecular lasers and QCL are powerful but not easily tunable. Photomixing is widely tunable but does not generate enough power for Schottky diodes. Frequency multiplication generates more power but has a limited tunability. Subharmonic mixing is also possible: a local oscillator in the cm- or the mm-range is used to pump a Schottky diode. Harmonics are generated inside the diode that beat with the THz signal to detect. In heterodyne detection, the NEP is limited by the bandwidth of the IF. It is then very sensitive for the detection of high spectral purity CW THz signals.

## 2. Photomixing Spectrometer

**Figure 1.**
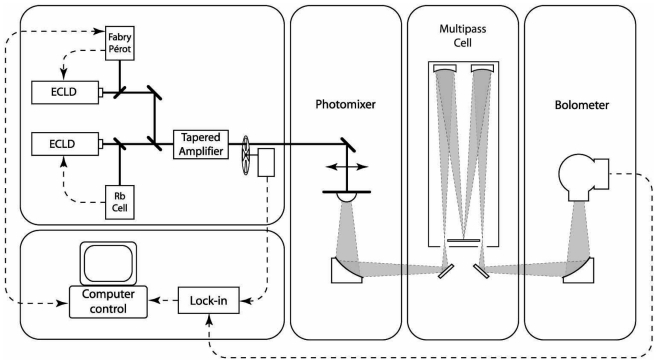
Photomixing spectrometer for high-resolution spectroscopy of gases.

Photomixing or optical heterodyne was first demonstrated by Brown *et al.* in 1993 [12] who used it to measure the self-broadening of SO_2_ [13]. An optical beatnote is generated by mixing two visible lasers and is tuned to the desired THz frequency. The two-color laser beam is focused onto a photomixer element which is composed of a semiconductor with a charge carrier lifetime in the region of ≈300 fs. The semiconductor response is sufficiently rapid to follow the THz beatnote frequency while not responding to the higher frequency mixing components. The THz frequency is therefore transferred to the electronic domain by the optically driven modulation of the photomixer conductivity. A current is generated by the application of an electric field and is coupled to a pair of antennae radiating continuous-wave monochromatic THz into free space. A spectrometer based on this source can be divided into the following functional units: a dual frequency optical source, a photomixer device, a THz beam path including sample cell, and a detector. The spectrometer constructed at the Laboratoire de Physico-Chimie de l'Atmosphère (LPCA) is illustrated in Figure 1 and includes a multi-pass sample cell and a liquid helium cooled Si bolometer detector.

### 2.1. Two Color Optical Source

The dual frequency optical source is composed of two lasers which are spatially overlapped with an identical polarization to form a two color beam. The choice of laser wavelength is governed by the absorption of the semiconductor material used in the photomixer. The most widely used material is low temperature grown (LTG) GaAs which absorbs strongly around 820 nm. Secondly the laser linewidth and tunability are critical as they define the spectral resolution and tuning range of the final instrument. Titanium Sapphire (Ti:Al_2_O_3_) lasers can provide a wide tuning range around 800 nm with an instantaneous line width in the order of 100 kHz, they do however need a complex tuning mechanism and frequency control system, require high power pump lasers, expert users and occupy a considerable volume. Nevertheless these lasers have provided excellent results when used for the cw-THz spectroscopy [14-16]. More recently the availability of extended cavity diode lasers (ECDL) along with tapered semiconductor optical amplifiers offers a more compact alternative, but is not available with an integrated frequency control system. Thankfully numerous frequency stabilization tools are available and can be adapted for this purpose. Although these lasers have a short term line width around 100 kHz at longer time scales they suffer from jitter broadening of the linewidth to around 10 MHz over a second. To optimize the discrimination power of cw-THz spectroscopy a spectral resolution of 1 MHz is required in the THz domain, hence each visible laser should have a linewidth not larger than 1 MHz over the timescale of a single measurement, typically one second. For a laser typically operating at around 374 THz this correspond to a stability of 10^−9^. Fortunately the atomic absorptions of Cesium and Rubidium provide convenient absolute frequency reference points around 852 nm and 780 nm, respectively. A servo loop can be implemented to lock a laser frequency to a saturated absorption feature ensuring an absolute frequency calibration and even a modest loop bandwidth allows linewidths of 1 MHz to be achieved. The second laser must be frequency swept to provide the spectrometer tunability, the difficulty is encountered with the conflicting requirements of a frequency stabilization scheme to achieve 1 MHz linewidth and the need to have a frequency reference that can be swept. One solution is a low-contrast Fabry-Perot interferometer. This type of instrument has relatively wide resonances which combined with the measurement of the transmitted and reflected beams of two path with a small angular variation provides relative frequency information at all frequencies. In this way the second laser can be frequency scanned with an active stabilization ensuring a linewidth in the region of 1 MHz. The difficulty with this solution is accuracy of the THz frequency generated, the spectral purity of the lasers ensures a good precision however the lack of absolute frequency information of the swept laser is only resolved by a standard wavelength meter with an accuracy of 50 MHz. In the case of the instrument that was realized at the Laboratoire de Physico-Chimie de l'Atmosphère the ECDL have an output power of 50 mW that is increased by a tapered semiconductor optical amplifier. The dual frequency beam is amplified to give access to a maximum power of 1 W. An alternative to this latter usual approach is to use a single laser oscillating with two modes [17,18]. In this way the perfect cancellation of any cavity instabilities is obtained allowing an excellent beatnote linewidth to be achieved, in the order of the 1 Hz but at the expense of tunability. Several bi-modes lasers have been implemented, often around 1,550 nm, and so this solution remains hampered by the lack of availability of photomixer devices operating at this wavelength, however the recent development UTC diode photomixers holds much promise [19,20].

### 2.2. Photomixer

A THz photomixer is a device composed of a fast photodetector connected to an antenna. The optical beating of the two optical frequencies υ_1_ and υ_2_ is converted by the photodetector to an AC voltage at a frequency equal to |υ_1_- υ_2_|. This voltage is applied to the antenna that radiates a THz wave in free-space. Two kinds of fast photodetectors are used in THz photomixers: photoconductors and photodiodes [16].

Photoconductors are particularly simple devices consisting of two metal electrodes patterned on a semi-insulating semiconductor. A DC voltage source is used to bias the device, its resistance is modulated at |υ_1_- υ_2_| and generates the THz current. The bandwidth is mainly limited by the photocarrier lifetime, to generate THz current lifetimes of the order of 0.1–10 ps are needed. The laser wavelength is generally between 0.7 and 1.6 μm depending on the semiconductor bandgap [21-23]. Photodiodes are generally based on an heterostructure of different semiconductors with different doping. In this case the bandwidth is limited by the transit-time of the carriers (electrons and holes) inside the structure. The best results at THz frequencies have been obtained with a uni-travelling carrier photodiode (UTC-PD) at 1.55 μm [19,20]. In this structure only electrons contribute to the bandwidth contrary to the *pin* photodiodes.

The most widely used antennas in THz photomixers are wideband in nature to preserve the large tunability that makes photomixing attractive for spectroscopy. Generally a bandwidth of at least one decade (for example: 300 GHz–3 THz) is required. Only a few antennas have such a wide bandwidth: spiral, log-periodic and horns. The two former are planar antennas that can be easily integrated with the photodetector. They unfortunately suffer from significant losses as the majority of the energy is radiated into the substrate and a silicon lens is needed to collect the THz wave. An alternative antenna that has recently been adopted for photomixer devices is the transverse-electromagnetic horn antenna (TEM-HA) which is a 3D structure that radiates directly in free-space without the need for a Si-lens [24].

**Figure 2.**
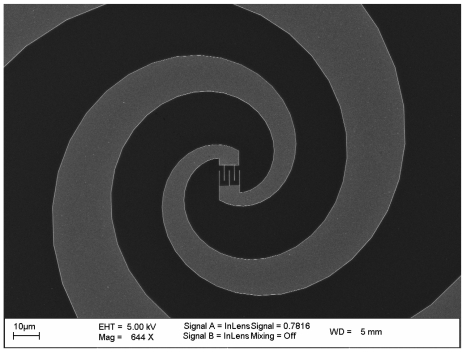
Scanning electron microscope image of the LTG-GaAs photomixer.

In the case of the LPCA spectrometer, the photomixer devices used are fabricated at the Institut d'Electronique de Microélectronique et de Nanotechnologie (IEMN). A layer of LTG-GaAs is grown by gas-source molecular beam epitaxy onto a standard semi-insulating GaAs substrate. The carrier lifetime may be measured by time-resolved photoreflectance yielding a typical value of 800 fs. The interdigitated photoconductor and the spiral antenna are defined by patterning a metal layer using electron beam lithography, Figure 2.

The width of the fingers is 0.2 μm and the active area of the photoconductor is 8 × 8 μm^2^. A Si_3_N_4_ layer is also deposited in order to reduce the optical reflection and to act as a passivation layer. The typical THz output power obtained with this type of photomixer is a few 100 nW at 1 THz for a photocurrent of 1–2 mA.

### 2.3. Instrument Sensitivity

**Figure 3.**
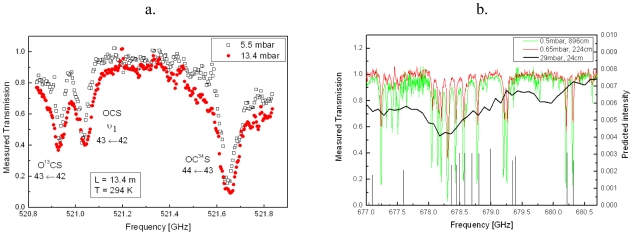
a.) Rotational transitions of OCS isotopologues, L = 13.4 m, T = 294 K.b.) Spectra of vinyl chloride at ambient temperature for various path lengths. Black line 24 cm. Red line 224 cm. Green line 896 cm. Black sticks indicate the predicted transition frequencies and relative intensities for the ^35^Cl isotopologue calculated from the spectroscopic parameters fitted elsewhere [28].

The application field of molecular spectroscopy depends strongly on the instrumental sensitivity: trace gas detection, probing of low-frequency ro-vibrational transitions or high J levels rotational transitions requires the measurement of weak molecular absorptions. The extension of the interaction distance between the radiation and the absorbing molecule has been very successfully exploited in the infrared with multiple pass sample cells and even resonant cavities. The difficulty encountered when trying to associate these techniques with a photomixing source is the highly divergent low power source beam [25,26]. Nevertheless careful characterization of the divergence of the THz beam combined with the calculation of beam diameter at all points along its trajectory have allowed a standard (Infrared Analysis, 35-V) multi-pass cell to be integrated into the spectrometer. This cell has been used at frequencies from 300 GHz to 1.7 THz offering a variable path length in the region of 2 m to 20 m with throughputs varying from 10% to 45%. An optical path length of 13.4 m allowed rotational transitions of the OC^34^S and O^13^CS isotopes to be observed (natural abundancies are 4.2% and 1.0%, respectively, Figure 3a).

The OC^34^S transition at 521.7 GHz is distorted by an intense OCS line at 522.6 GHz. The O^13^CS transition is observed at 520.9 GHz and the line at 521.0 GHz is the excited symmetric stretching ν_1_ mode. The O^13^CS line has a tabulated intensity of 2 × 10^−23^ cm^−1^/(molecule.cm^−2^), indicating that for pure gases the spectrometer is capable of resolving line intensities as weak as 10^−25^ cm^−1^/(molecule.cm^−2^). The sensitivity is also demonstrated by the pure rotational spectra of vinyl chloride (C_2_H_3_Cl) for various path lengths (Figure 3b) [27]. A single pass with a path length of 24 cm required a pressure of 29 mbar to obtain a reasonable absorption signal, at this pressure no individual lines can be resolved. As the path length is increased lower gas pressures can be used and the lines can be resolved, increasing from 224 cm to 896 cm reveals further transitions.

This spectrum is a result of the normalization of two frequency scans, one in the presence of the target molecule and the second with an empty measurements cell. This technique allows the removal of any systematic non-molecular signals such as Fabry Pérot etalons. The recording of two identical scans enables the quality of this normalization procedure to be assessed and the measurement of the minimum absorption and hence spectrometer sensitivity to be identified. A path length of 13.44 m yielded a minimum absorption of α_min_ = 2 × 10^−5^ cm^−1^, compared to a value of 10^−8^ cm^−1^ for an instrument limited only by the detector NEP and the source power [29].

### 2.4. Frequency Metrology

Standard laser stabilization techniques can easily provide a spectral purity of 1 MHz, in the case of a scanning Fabry Pérot etalon this is directly transferred to the frequency precision of the photomixing spectrometer. This kind of instrument has already shown its utility for absorption profile analysis but suffers from poor frequency accuracy and so cannot be used to determine molecular transition frequencies. To overcome this difficulty a reliable frequency reference is required. One solution is to lock both lasers onto two different modes of a single high finesse Fabry-Pérot cavity. This has the advantage of allowing locking techniques like Pound-Drever-Hall to be used reducing the ECLD linewidths to less than 100 kHz [30]. More importantly the use of a single etalon significantly reduces the system sensitivity to environmental influences like temperature, the frequency of the THz radiation is an integer multiple of the free spectral range (FSR) of the cavity. The disadvantages are that the FSR must be known with an excellent accuracy and that the THz frequency cannot be tuned. Two different methods have been proposed to overcome the lack of tunability. A third laser can be phase locked to a laser itself locked to the etalon, then frequency swept by tuning the oscillator in the phase lock loop. This type of system has successfully been applied to distributed Bragg reflector lasers yielding a final tuning range of 3 GHz and an accuracy of 10^−7^ [31]. The second method that has been demonstrated uses acousto-optic modulators to provide a tuning range in the order of 10 MHz [32].

An alternative frequency reference that has recently emerged is a frequency comb that has the advantages of offering a stable reference with an excellent accuracy over a large range of frequencies. A train of short pulses from a mode locked laser generates the frequency comb, the length of the laser cavity and hence the repetition rate is stabilized by a phase locked loop (PLL) and can be easily referenced to a primary frequency source such as an atomic clock or a GPS signal. The frequency of an individual mode of the frequency comb is given by its mode number and the carrier envelope offset (1) that results from of the difference in phase and group velocity of the laser cavity [33]:

(1) $$f_{n}=n.f_{\text{rep}}+f_{ce}$$

**Figure 4.**
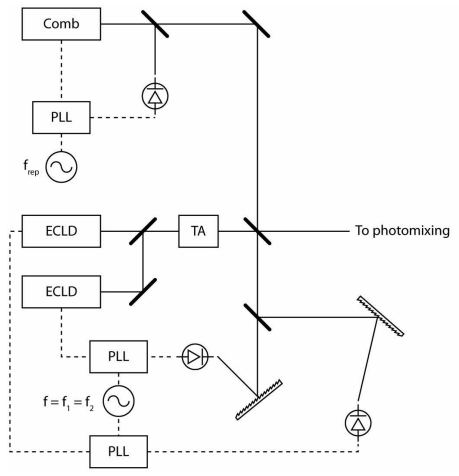
The frequency comb is mixed with the two-color beam each of the ECDLs are phase locked to the nearest mode of the frequency comb synthesizing the difference frequency between the ECDLs with an accuracy of 10^−8^.

**Figure 5.**
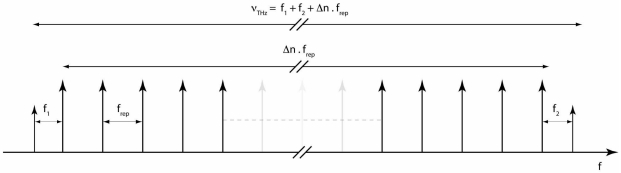
Optical frequency diagram containing the regularly spaced frequency comb modes and the two ECLD modes. Note that the lower frequency ECLD is locked to the low frequency side of the FC mode whereas the higher frequency ECLD is locked to the high side.

In the context of a photomixing instrument the source lasers can be phase locked to two different modes resulting in a difference frequency independent of the carrier offset. The two-color source is mixed with the frequency comb and dispersed by a grating allowing a small photodiode to select the optical frequencies corresponding to one of the cw lasers and a small number of frequency comb modes (Figure 4). The beatnote between the cw laser and the nearest frequency comb mode is phase locked to an electronic oscillator. In this manner both lasers are coherently locked to the frequency comb and the quality of the THz frequency synthesized depends on the stability and accuracy of the laser repetition rate (Figure 5). Measurement of the beatnote between the FC and the ECDL by a spectrum analyzer indicates a FWHM of approximately 100 kHz for an integration time of 100 ms.

In order to adapt this technique for the construction of a spectrometer a scanning mechanism is required the simplest approach is to slowly adjust the repetition rate of the frequency comb. This presents the disadvantage that as the mode separation increases the absolute frequency of a given mode increases rapidly, for example a pair of ECDL operating at 800 nm with a gain bandwidth of 10 GHz can provide a tuning range of only 2.5 MHz at 1 THz. To overcome this difficulty the beatnote frequencies of the two source lasers can be scanned, hence the frequency comb remains fixed. Although this strategy has proven successful for scans lengths of up to 10 MHz extending it significantly beyond this is not practical due to the electronic filtering required to isolate the beatnote between a single frequency comb mode and a cw laser. To examine the performance of this system a number of well known molecular transitions were measured, the J = 67 ← 66 line of OCS at 813 GHz is suitable candidate as it has an intense THz signature and is one of the highest frequency transitions that has already benefited from position measurement with an accuracy of 60 kHz [34]. At a frequency of 813,353.733 MHz the value measured with the frequency comb photomixing spectrometer is within 30 kHz of the previous measurement.

## 3. Application to Gas Phase Spectroscopy

Pure rotational transitions of small polar compounds and the vibrational lines of low-frequency motions are located in the THz frequency domain. The sensitivity and spectral purity of cw-THz photomixing spectroscopy initially limited its application to pure rotational transitions of highly polar molecules such as hydracids, carbon sulphide, hydrogen sulphide and ammonia [29,35,36]. However, a larger variety of molecular transitions may be probed in the THz frequency range but their spectroscopic analysis encounters both theoretical and experimental difficulties: due to the internal rotation of methyl groups, the THz spectra of methanol, toluene or acetone present a high density of rotational lines and their assignment requires specific molecular Hamiltonians [37]. The skeletal deformation of polycyclic aromatic hydrocarbons (PAH) [38] as well as large amplitude motion of bio-molecules [39] are also observed in the THz frequency range but they both requires a highly sensitive instruments due to their low volatility and the weakness of the rovibrational line intensities. Moreover, due to the unique selectivity of the THz radiation, the conformational landscape of these highly flexible molecules has to be taken into account for the understanding of the THz spectra [40].

Generally for the analysis of gases an instrument capable of resolving transitions with a quality factor in the order of 10^6^ is required, for example at 1 THz HCN has a Doppler width of 1 MHz FWHM at ambient temperature. The collisional broadening is around 3 MHz/mbar, hence an instrument with a spectral purity in the same order of magnitude is able to exploit the fineness of the transitions at low pressure to have an excellent discrimination between multiple species and provide a calibration free quantification at pressures above the Doppler limit. The advantages offered by the use of THz frequencies are the ability to make quantitative measurements in the presence of aerosols and a high level of discrimination between chemical species. At present there are only a small number of laboratories that are capable of undertaking high resolution THz spectroscopy above 1 THz. Among the techniques in this range photomixing does not require access to the frequency multiplication components and it is a reasonably modest installation compared to a synchrotron. Its disadvantage is the weak power level produced, around 1 μW at 1 THz, that necessitates the use of a sensitive detection system.

### 3.1. Detection of Atmospheric Compounds

**Figure 6.**
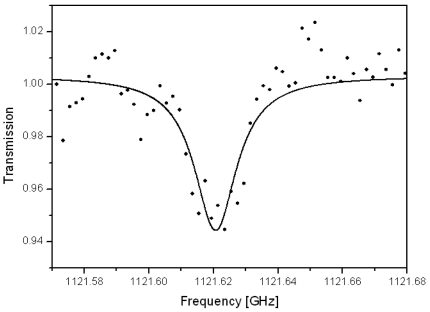
Measured spectrum of cigarette smoke at a pressure of 2 mbar, a transition of HCOOH is observed with a path length of 20.16 m. Line intensity = 2.2 × 10^−22^ cm^−1^/(molecule·cm^−2^). The measured datapoints (filled points) are fitted with a Lorentzian absorption profile (solid line).

The THz waveband is particularly rich in molecular transitions so it can provide the opportunity to measure many gas phase species. As the optical absorption is recorded the concentration can be easily determined using the transition intensity, the measurement is therefore direct and does not depend on a calibration procedure. At low pressure the absorption lines are narrow hence excellent discrimination between chemical species can be achieved allowing the simultaneous monitoring of multiple species. The relatively long wavelength of the radiation permits samples, such as smoke, that are diffusive in the optical domain to be successfully probed. Although THz radiation has many attractive features for the analysis of industrial emissions the associated instrumentation is presently delicate and requires the controlled environment of a laboratory. Cigarette smoke is an ideal subject to demonstrate the potential of cw-THz as it can be easily generated, contains a large variety of compounds and aerosols. The use of a multiple pass cell providing path lengths up to 31 m allowed the chemical species in Table 1 to be identified and quantified in a sample of mainstream cigarette smoke, including for the first time to our knowledge the measurement of formic acid in the THz range, a typical spectrum for which is given in Figure 6.

To ensure a correct molecular identification multiple transitions were recorded at various pressures. A Lorentzian absorption profile is applied to determine the integrated line intensity and hence the species concentration. The detection limit is estimated for an absorption with a unity signal to noise ratio when compared to an identical measurement of an empty sample cell. At the present time the number of species that may be detected is limited only by the sensitivity of the instrument. The simultaneous measurement of multiple species is simply dependent on the continuous tunning range of the lasers used. Unlike ECDL that have a continuous tunning range of 10 GHz the use of a Distributed FeedBack (DFB) diode laser allows a rapid tunability of around 1,000 GHz to be achieved and so is an ideal choice for the surveillance of multiple species.

DiMethyl SulfOxyde (DMSO) is volatile organic compound (VOC) which is not only a mimic of the chemical warfare agent mustard gas, but also a solvent employed in certain biochemical procedures. Its detection in the gas phase presents both military and civilian applications. Unlike the other gases studied and despite being classified as a VOC under standard conditions, DMSO is in the liquid state. It presents a vapor pressure of only 0.56 mbar, its measurement here was conducted on a sample cell that is close to the saturation point of DMSO. Due to the lack of existing THz data for this molecule in the gas phase microwave measurements at frequencies up to 30 GHz form the starting point for its analysis by high resolution ro-vibrational spectroscopy [41]. The refinement of its spectroscopic parameters used in the microwave band and the addition of supplementary parameters will eventually allow its THz spectrum to be accurately predicted. This process requires the measurement of the center frequency of many transitions with an accuracy approaching that which can be achieved in the microwave region, *i.e.*, 50 kHz or better. Its measurement at a synchrotron facility with a resolution of 150 MHz is insufficient to completely resolve the rotational structure of this asymmetric molecule characterized by a high density of rotational lines.

**Figure 7.**
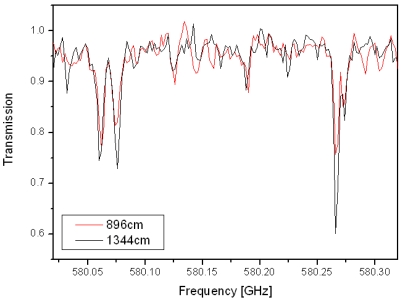
Transmission spectra of DMSO at ambient temperature, with a pressure of 0.1 mbar, for path lengths of 896 cm (red) and 1,344 cm (black).

The resolution offered by the photomixing source has allowed some of the most intense rotational lines of the DMSO to be measured (Figure 7), although without the frequency metrology scheme presented in section 2.4, hence the line positions suffer from an absolute accuracy in the region of 50 MHz and can not be used to refine the parameters. The combination of the multipass cell and the frequency metrology will provide an instrument with sufficient sensitivity and accuracy to pursue this topic.

### 3.2. Determination of Spectroscopic Parameters—Line Profile Analysis (HCN)

**Figure 8.**
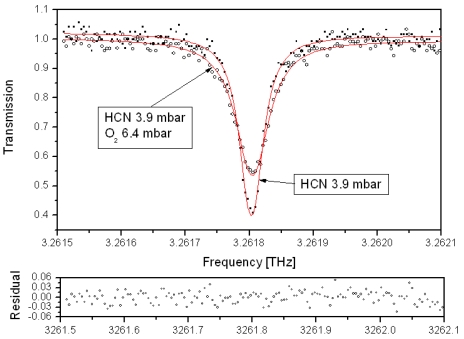
Transmission spectra of HCN at ambient temperature for J = 37 ←36. Red line fitted absorption profile.

The large tunability and good spectral resolution of the photomixing instrument ensures that it is an ideal for the measurement of absorption profiles of the small linear molecules with a large permanent dipole moment. Hydrogen cyanide is present in comets, interstellar clouds, and in the atmospheres of several planets and moons like Titan [42,43]. Knowledge of its spectral parameters is important for star forming models as well as for monitoring industrial pollution [35]. The transition profile parameters are therefore tabulated in many spectroscopic databases such as HITRAN 2004 [44] where the air broadening coefficients for the pure rotational transitions J ≤ 29 are given by a polynomial based on measurements of parallel bands in the infrared [45,46], for values above J = 29 a constant value is applied due to the lack of experimental data. The photomixing spectrometer can however directly measure the absorption profiles of these pure rotational transitions, an example of the J = 37 ← 36 line at 3.26 THz is given in Figure 8 and represents the highest operational frequency of the instrument.

**Figure 9.**
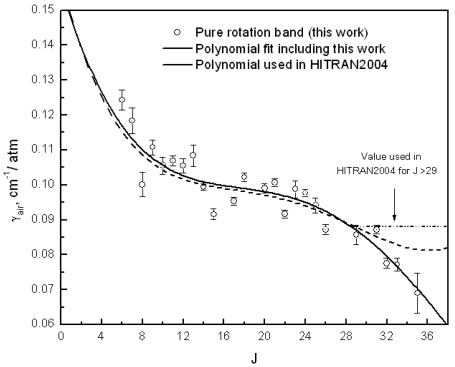
Air broadening coefficient of HCN measured over the frequency range 530 GHz to 3.3 THz. Measured values (open points), HITRAN 2004 polynomial (dotted line), updated polynomial for HITRAN 2008 (solid line).

The collisional broadening of the lines at various pressures by oxygen and nitrogen allows the linewidth to be extracted from a fitted Voigt profile and the broadening coefficient determined. The air broadening coefficient is then calculated using the nitrogen and oxygen contributions. In this manner all the transitions from J = 5 to J = 36 (532 GHz to 3.26 THz) have been characterized [35]. The values up to J = 25 show a good agreement with previous work and the tabulated values. At high frequency a large difference is noted as the broadening coefficient continues to decrease, Figure 9. These knew measurement have been added to the existing data to generate an updated polynomial for HITRAN 2008 [47] release applicable for values up to J = 40.

## 7. Conclusions

The large tunability and spectral purity of the photomixing source is ideal for the measurement of THz absorption profiles of volatile polar molecules, in particular over the frequency interval 1 THz to 3 THz where few alternatives can rival its performance. The potential of this instrument has been extended by increasing its sensitivity and adding a mechanism by which the THz frequency is accurately synthesized. The ability to measure weakly absorbing transitions has allowed a number of gas phase species to be quantified in a sample of unfiltered cigarette smoke, demonstrating the utility of THz radiation to probe normally diffuse media. Several transitions of two VOC molecules have also been resolved indicating that the THz frequency synthesizer will now unlock the spectroscopy of these larger molecules by the progressive refinement of their parameters with the position data at THz frequencies. The principal perspectives for the photomixing technique for the study of gas phase subjects are oriented in two directions. In the near term, high resolution laboratory based spectroscopy of molecular systems using technology around 800 nm and providing detailed molecular information for astrophysics or satellite data interpretation. In the longer term, simpler ‘light weight’ or maybe portable systems benefiting from the excellent choice of lasers at 1,550 nm that can meet the needs of industrial applications such as emission monitoring or trace detection.

## Figures and Tables

**Table 1.** Measured concentration of species contained in the cigarette smoke.

**Molecule**	**Concentration**	**Detection limit**
HCN	73 ppm	0.2 ppm
HCOOH	29 ppm	2 ppm
H2CO	37 ppm	3 ppm
CO	1030 ppm	14 ppm
NO	43 ppm	1.9 ppm
